# Supplementary material for: Evaluation of Blood Tumor Mutation Burden for the Efficacy of Second-Line Atezolizumab Treatment in Non-Small Cell Lung Cancer: BUDDY Trial
Source: Cells. 2023 Apr 25;12(9):1246. doi: 10.3390/cells12091246 (PMC10177441; doi:10.3390/cells12091246)
Supplement: Supplementary file 1 [file cells-12-01246-s001.zip › SM/cells-2308753-SM.pdf]

Supplementary Table S1. Baseline characteristics of enrolled patients.

Characteristics	No. of Patients, *n* = 100
Metastatic sites, total	1.0 (0-4)
Contralateral lung	39
Pleura / Pleural effusion	35
Pericardial effusion	4
Liver	7
Adrenal gland	8
Brain	6
Bone	19
Extrathoracic lymph node	26
Others [1]	10
Comorbidities: Yes / No	51 / 49
Myocardial infarction	1
Cerebrovascular accident	3
COPD	21
Asthma	2
PF-ILD	1
Connective tissue disease [2]	2
Peptic ulcer disease	1
Chronic liver disease [3]	2
Diabetes mellutis	27
Chronic kidney disease	4
Prior solid tumor [4]	3
Laboratory findings	
WBC, x10^3/uL	7.80 (3.20-24.07)
ANC, x10^3/uL	4.84 (1.46-21.78)
Lymphocyte, x10^3/uL	1.69 (0.18-4.32)
Monocyte, x10^3/uL	0.74 (0.05-1.78)
Eosinophil, x10^3/uL	0.12 (0.00-1.58)
Hemoglobin, g/dL	11.65 (8.20-16.10)
Platelet, x10^3/uL	259 (93-786)
NLR	2.84 (0.83-24.04)
dNLR	1.80 (0.67-10.90)
PLR	158.15 (37.30-3138.57)
LDH, IU/L	268 (131-843)
T3, ng/mL	1.14 (0.51-162.20)
Free T4, ng/dL	1.20 (0.85-1.80)
TSH, uIU/mL	1.58 (0.00-11.48)
Hypo- / Hyper-thyroidism	0 / 0
Subclinical hypo- / hyper-thyroidism	4 / 5
EGFR mutation, tested	80 (100.0)
Positive	11 (13.8)
Ex19del	3 (3.8)
L858R	2 (2.5)
Ex19del + T790M	2 (2.5)
L858R + T790M	1 (1.3)

T790M	1 (1.3)
Others [5]	1 (1.3)
Unknown	1 (1.3)
Wild type	69 (86.3)
Prior antibiotics treatment	18
IV / PO	7 / 11
Within 90 days: Yes / No	8 / 10
Prior steroid treatment	7
IV / PO	1 / 6
Within 90 days: Yes / No	3 / 4
Prior surgery	15
Location: Lung / Others [6]	14 / 1
Wedge resection	4
Lobectomy	9
Bilobectomy	1
Prior RT (last)	43
Location	
Lung	30
Brain	6
Lymph node	2
Bone	4
Others [7]	1
Dose, cGy	6000 (1500-6625)
Last RT to atezolizumab, months	6.5 (0.1-53.5)
Post-PD subsequent chemotherapy, n (%)	46 (100.0)
Regimen	
Platinum doublet	7 (15.2)
Cytotoxic monotherapy	32 (69.6)
Tyrosine kinase inhibitor	5 (10.9)
Others [8]	2 (4.3)
Best response	
CR	0 (0.0)
PR	8 (17.4)
SD	18 (39.1)
PD	11 (23.9)
NE	9 (19.6)
Objective response rate, %	8 (17.4)
PFS2 [9], months (95% CI)	7.5 (5.1-9.8)
Concurrent radiotherapy	7
Location	
Lung	2
Brain	3
Bone	3
Others [10]	1

Values are presented as median (range) or number (%). [1] chest walls (*n* = 3), diaphragm (*n* = 1), abdominal cavity (*n* = 1), kidney (*n* = 2), thigh (*n* = 1), unknown (*n* = 2). [2] rheumatoid arthritis (*n* = 1), fibromyalgia (*n* = 1). [3] chronic hepatitis B (*n* = 2). [4] small cell lung cancer (*n* = 1), prostate cancer (*n* = 1), thyroid cancer (*n* = 1). [5] exon 20 S784F (*n* = 1). [6] nephrectomy (*n* = 1). [7] abdomen (*n* = 1). [8] atezolizumab (*n* = 1) and ramucirumab (*n* = 1). [9] PFS2 refers to time

from the first day of atezolizumab treatment to the day of objective disease progression of the subsequent therapy or death. [10] chest walls (n = 1). COPD, chronic obstructive pulmonary disorder; PF-ILD, progressive fibrosing phenotype of interstitial lung disease; WBC, white blood cell; ANC, absolute neutrophil count; NLR, neutrophil-to-lymphocyte ratio; dNLR, derived neutrophil-to-lymphocyte ratio; PLR, platelet-to-lymphocyte ratio; LDH, lactate dehydrogenase; TSH, thyroid stimulating hormone; EGFR, epidermal growth factor receptor; IV, intravenous; PO, per oral; RT, radiotherapy; PD, progressive disease; CR, complete response; PR, partial response; SD, stable disease; NE, not evaluable; PFS, progression-free survival.

Supplementary Table S2. Comparison of baseline characteristics according to best response and clinical benefit rate.

Characteristics	Non-PR (n=90)	PR (n=10)	P-value	NDB (n=75)	DCB (n=25)	*P*-value
Age						
< 65years	41 (45.6)	3 (30.0)	0.506	33 (44.0)	11 (44.0)	1.000
≥ 65years	49 (54.4)	7 (70.0)		42 (56.0)	14 (56.0)	
Sex						
Female	16 (17.8)	0 (0.0)	0.358	14 (18.7)	2 (8.0)	0.345
Male	74 (82.2)	10 (100.0)		61 (81.3)	23 (92.0)	
Smoking						
Never smoker	21 (23.3)	0 (0.0)	0.115	18 (24.0)	3 (12.0)	0.202
Ever smoker	69 (76.7)	10 (100.0)		57 (76.0)	22 (88.0)	
Histology						
SqCC	37 (45.1)	2 (22.2)	0.291	32 (47.1)	7 (30.4)	0.164
ADC	45 (54.9)	7 (77.8)		36 (52.9)	16 (69.6)	
Liver metastasis	7 (7.8)	0 (0.0)	1.000	7 (9.3)	0 (0.0)	0.187
Brain metastasis	5 (5.6)	1 (10.0)	0.478	5 (6.7)	1 (4.0)	1.000
Comorbidities						
No	46 (51.1)	3 (30.0)	0.319	38 (50.7)	11 (44.0)	0.564
Yes	44 (48.9)	7 (70.0)		37 (49.3)	14 (56.0)	
EGFR mutation: Yes	11 (15.3)	0 (0.0)	0.591	11 (18.0)	0 (0.0)	0.058
ALK rearrangement: Yes	1 (1.5)	0 (0.0)	1.000	1 (1.9)	0 (0.0)	1.000
PD-L1 (22C3)						
TPS <50%	40 (65.6)	1 (16.7)	0.029	34 (66.7)	7 (43.8)	0.101
TPS ≥50%	21 (34.4)	5 (83.3)		17 (33.3)	9 (56.3)	
PD-L1 (SP263)						
TPS <50%	51 (67.1)	4 (50.0)	0.438	42 (66.7)	13 (61.9)	0.691
TPS ≥50%	25 (32.9)	4 (50.0)		21 (33.3)	8 (38.1)	
PD-L1 (SP142, TC)						
TPS <5%	12 (85.7)	1 (100.0)	1.000	10 (90.9)	3 (75.0)	0.476
TPS ≥5%	2 (14.3)	0 (0.0)		1 (9.1)	1 (25.0)	
PD-L1, high expression (≥50% or ≥5%)						
No	57 (66.3)	3 (33.3)	0.071	49 (67.1)	11 (50.0)	0.144
Yes	29 (33.7)	6 (66.7)		24 (32.9)	11 (50.0)	
NLR, median						
<2.84	45 (50.0)	5 (50.0)	1.000	35 (46.7)	15 (60.0)	0.248
≥2.84	45 (50.0)	5 (50.0)		40 (53.3)	10 (40.0)	
dNLR, median						
<1.80	45 (50.0)	5 (50.0)	1.000	36 (48.0)	14 (56.0)	0.488
≥1.80	45 (50.0)	5 (50.0)		39 (52.0)	11 (44.0)	
PLR, median						
<158.15	45 (50.0)	5 (50.0)	1.000	35 (46.7)	15 (60.0)	0.248
≥158.15	45 (50.0)	5 (50.0)		40 (53.3)	10 (40.0)	
Prior antibiotics						
<90days	8 (44.4)	0 (0.0)	-	7 (50.0)	1 (25.0)	0.588
≥90days	10 (55.6)	0 (0.0)		7 (50.0)	3 (75.0)	

Prior steroids						
<90days	4 (66.7)	0 (0.0)	0.429	4 (66.7)	0 (0.0)	0.429
≥90days	2 (33.3)	1 (100.0)		2 (33.3)	1 (100.0)	

Values are presented as median (range) or number (%). PR, partial response; NDB, non-durable benefit; DCB, durable clinical benefit; SqCC, squamous cell carcinoma; ADC, adenocarcinoma; EGFR, epidermal growth factor receptor; ALK, anaplastic lymphoma kinase; PD-L1, programmed death-ligand 1; TPS, tumor proportional score; TC, tumor cell; NLR, neutrophil-to-lymphocyte ratio; dNLR, derived neutrophil-to-lymphocyte ratio; PLR, platelet-to-lymphocyte ratio.

Supplementary Table S3. The safety profile in SEP.

Adverse events (AEs)	No. of Patients, *n* = 100				
AESI [1]	2				
SAE	27				
AEs leading to interventions					
Withdrawal	14				
Interruption	23				
Dose reduction	0				
**Adverse events (AEs)**	**All Gr**	**Gr 1**	**Gr 2**	**Gr 3**	**Gr 4-5** [2]
Any AEs	82	57	39	16	12
Dyspnea	22	9	11	0	1 [3]
Anorexia	14	5	9	0	0
Pain, cancer-related	11	3	5	3	0
Sputum	10	6	4	0	0
Fever	8	6	1	1	0
Chest discomfort	7	5	2	0	0
Constipation	7	2	5	0	0
Cough	7	5	2	0	0
Generalized muscle weakness	7	1	4	2	0
LDH increased	6	5	1	0	0
Diarrhea	6	1	4	1	0
Edema	6	4	1	1	0
Paresthesia	6	5	1	0	0
Pneumonia	6	1	0	3	2 [3]
Musculoskeletal pain	5	4	1	0	0
Dizziness	3	2	1	0	0
Headache	3	3	0	0	0
Hemoptysis	3	2	0	1	0
Hyperkalemia	3	1	0	1	1 [3]
Pneumonitis	3	1	0	1	1 [3]
Sudden (cardiac) death	3	0	0	0	3 [4]
Abdominal pain	2	2	0	0	0
Back pain	2	2	0	0	0
Delirium	2	1	0	1	0
Depression	2	0	2	0	0
Dyspepsia	2	2	0	0	0
Dysuria	2	1	1	0	0
Fracture	2	0	0	2	0
Hoarseness	2	2	0	0	0
Hypercalcemia	2	0	0	0	2 [3]
Insomnia	2	0	2	0	0
Azotemia	1	1	0	0	0
Benign prostatic hyperplasia	1	1	0	0	0
Blurred vision	1	0	1	0	0
Brain atrophy	1	1	0	0	0
Breast pain	1	1	0	0	0
Chills	1	1	0	0	0

Cholecystitis	1	0	0	1	0
Condition aggravated	1	0	1	0	0
CRP increased	1	1	0	0	0
Creatinine increased	1	1	0	0	0
Dermatitis	1	1	0	0	0
Diplegia	1	1	0	0	0
Gait disturbance	1	0	1	0	0
Gout	1	0	1	0	0
Hematochezia	1	1	0	0	0
Hematuria	1	0	1	0	0
Hyperhidrosis	1	0	1	0	0
Nausea	1	0	1	0	0
Pericardial effusion	1	0	0	0	1 [3]
Platelet count decreased	1	1	0	0	0
Pulmonary embolism	1	0	1	0	0
Seizure	1	0	0	1	0
Sepsis	1	0	0	0	1 [3]
Tinnitus	1	1	0	0	0
Transient ischemic attack	1	0	0	1	0
Vomiting	1	0	1	0	0
Weight loss	1	1	0	0	0
Wheezing	1	1	0	0	0
**Suspicious immune-related AEs (irAEs)**	**All Gr**	**Gr 1**	**Gr 2**	**Gr 3**	**Gr 4-5**
Any irAEs	41	28	18	2	0
Hepatitis (LFT elevation)	15	12	1	2	0
Pruritus	13	7	6	0	0
Myalgia	8	4	4	0	0
Fatigue	7	4	3	0	0
Rash	7	4	3	0	0
Hypothyroidism	5	4	1	0	0
Hyperthyroidism	2	1	1	0	0
Adrenal insufficiency	1	0	1	0	0
Arthralgia	1	0	1	0	0
Flu-like symptom	1	1	0	0	0

Values are presented as numbers. [1] hypercalcemia (n = 1), dyspnea (n = 1). [2] grade 4 (n = 1) and 5 (n = 11). [3] grade 5. [4] grade 4 (n = 1) and 5 (n = 2). SEP, safety-evaluable population; AESI, adverse events with special interest; SAE, serious adverse event; Gr, grade; LFT, liver function test.

Supplementary Table S4. Multivariate Cox proportional analyses for PFS and OS in cfDNA BEP (*n* = 86).

Characteristic	PFS		OS	
	**Multivariate, HR (95% CI)**	**P-value**	**Multivariate, HR (95% CI)**	***P*-value**
Sex		0.092	-	-
Female	1 (ref)			
Male	0.33 (0.09-1.20)			
Histology	-	-		0.083
Adenocarcinoma			1 (ref)	
Squamous cell carcinoma			2.04 (0.91-4.57)	
Liver metastasis	NA	-	NA	-
No				
Yes		-		
EGFR mutation		0.831	-	-
No	1 (ref)			
Yes	0.83 (0.16-4.44)			
PD-L1, high expression		0.766	-	-
No	1 (ref)			
Yes	0.82 (0.23-2.98)			
bTMB from C0 to C4		0.005	-	-
Decreased or No change	1 (ref)			
Increased	4.95 (1.63-15.07)			
cfDNA at C0		0.003		0.396
<8.6 ng/mL	1 (ref)		1 (ref)	
≥8.6 ng/mL	5.16 (1.73-15.37)	-	1.43 (0.62-3.30)	
hVAF at C0	-	-		0.614
<3.9%			1 (ref)	
≥3.9%			1.53 (0.30-7.89)	
hVAF from C0 to C4		0.453	-	-
Decreased or No change	1 (ref)			
Increased	2.58 (0.22-30.88_			
VAFSD at C0	-	-		0.013
<0.014			1 (ref)	
≥0.014			2.84 (1.24-6.50)	
VAFSD from C0 to C4		0.236	-	-
Decreased or No change	1 (ref)			
Increased	0.36 (0.07-1.95)	-		

PFS, progression-free survival; OS, overall survival; BEP, biomarker-evaluable population; HR, hazard ratio; CI, confidence interval; NA, not available; EGFR, epidermal growth factor receptor; PD-L1, programmed death-ligand 1, bTMB, blood tumor mutation burden; cfDNA, circulating cell-free DNA; hVAF, highest variant allele frequency; SD, standard deviation.
