# Supplementary material for: Evaluation of Blood Tumor Mutation Burden for the Efficacy of Second-Line Atezolizumab Treatment in Non-Small Cell Lung Cancer: BUDDY Trial
Source: Cells. 2023 Apr 25;12(9):1246. doi: 10.3390/cells12091246 (PMC10177441; doi:10.3390/cells12091246)
Supplement: Supplementary file 1 [file cells-12-01246-s001.zip › SM/Supplementary figures.pdf]

Fig. S1a

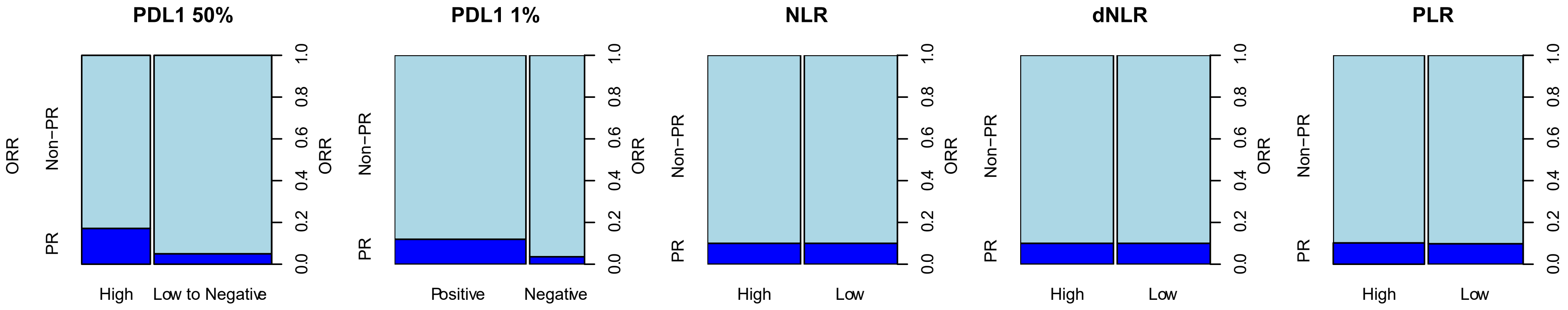
PDL1 50%
PDL1 1%
NLR
dNLR
PLR
ORR
Non−PR
PR
0.0
0.2
0.4
0.6
0.8
1.0
High
Low to Negative
Positive
Negative
High
Low
High
Low
High
Low

Fig. S1b

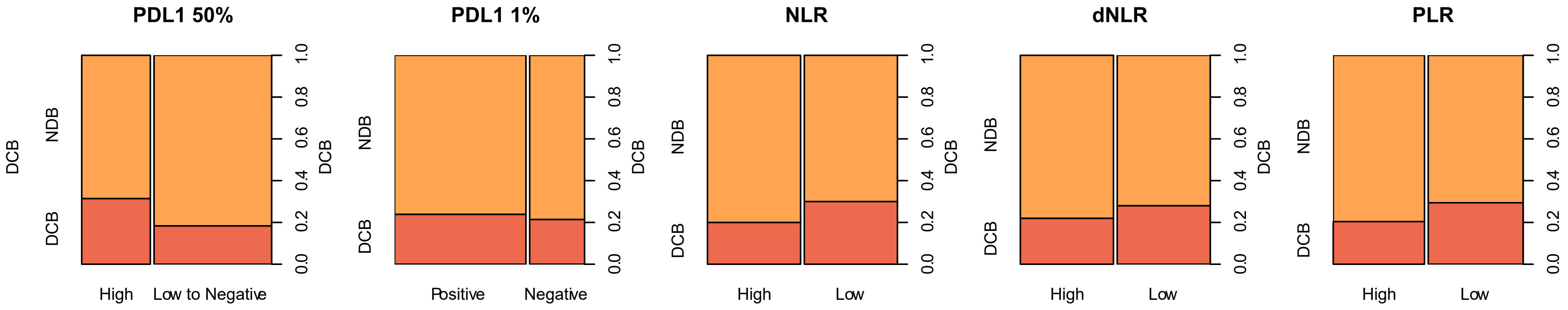

PDL1 50%
PDL1 1%
NLR
dNLR
PLR
DCB
NDB
DCB
0.0
0.2
0.4
0.6
0.8
1.0
High
Low to Negative
Positive
Negative
Low

Fig. S1c

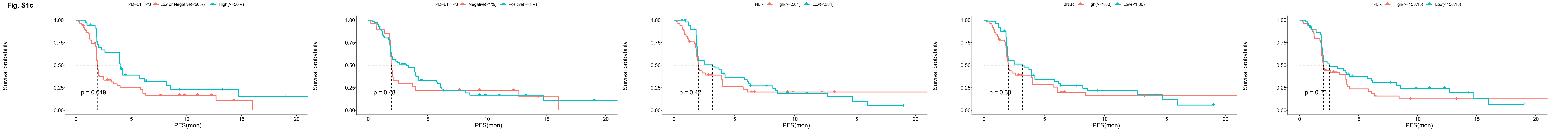

PD−L1 TPS	0	5	10	15	20
Low or Negative(<50%)	60	12	5	1	0
High(>=50%)	35	11	4	2	1

PD−L1 TPS	0	5	10	15	20
Negative(<1%)	28	6	4	1	0
Positive(>=1%)	67	17	5	2	1

NLR	0	5	10	15	20
High(>=2.84)	50	9	3	1	1
Low(<2.84)	50	16	6	2	0

dNLR	0	5	10	15	20
High(>=1.80)	50	10	3	1	1
Low(<1.80)	50	15	6	2	0

PLR	0	5	10	15	20
High(>=158.15)	49	9	2	1	1
Low(<158.15)	51	16	7	2	0

PFS(mon)

Fig. S1d

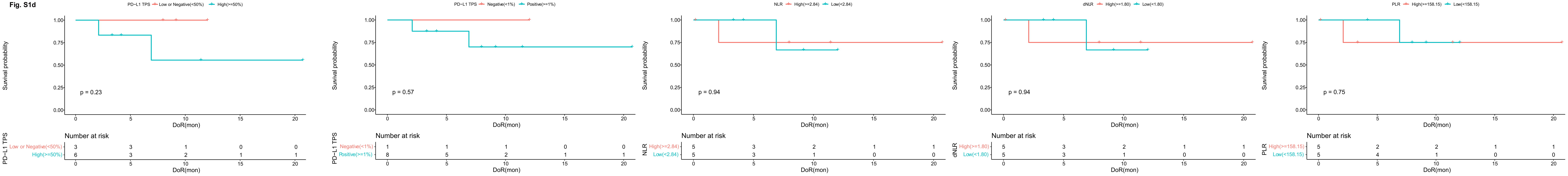
PD−L1 TPS Low or Negative(<50%) High(>=50%)
Survival probability
p = 0.23
DoR(mon)
Number at risk
Low or Negative(<50%) 3 3 1 0 0
High(>=50%) 6 3 2 1 1
PD−L1 TPS Negative(<1%) Positive(>=1%)
p = 0.57
Negative(<1%) 1 1 1 0 0
Positive(>=1%) 8 5 2 1 1
NLR High(>=2.84) Low(<2.84)
p = 0.94
High(>=2.84) 5 3 2 1 1
Low(<2.84) 5 3 1 0 0
dNLR High(>=1.80) Low(<1.80)
p = 0.94
High(>=1.80) 5 3 2 1 1
Low(<1.80) 5 3 1 0 0
PLR High(>=158.15) Low(<158.15)
p = 0.75
High(>=158.15) 5 2 2 1 1
Low(<158.15) 5 4 1 0 0

Fig. S1e

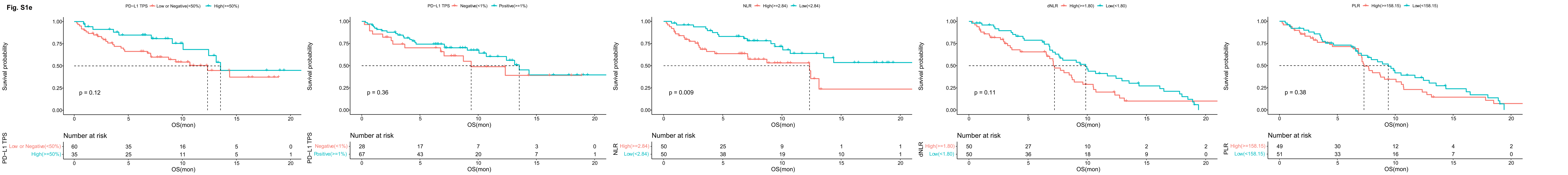
PD−L1 TPS Low or Negative(<50%) High(>=50%)
p = 0.12
Survival probability
OS(mon)
Number at risk
Low or Negative(<50%) 60 35 16 5 0
High(>=50%) 35 25 11 5 1
PD−L1 TPS Negative(<1%) Positive(>=1%)
p = 0.36
Negative(<1%) 28 17 7 3 0
Positive(>=1%) 67 43 20 7 1
NLR High(>=2.84) Low(<2.84)
p = 0.009
High(>=2.84) 50 25 9 1 1
Low(<2.84) 50 38 19 10 1
dNLR High(>=1.80) Low(<1.80)
p = 0.11
High(>=1.80) 50 27 10 2 2
Low(<1.80) 50 36 18 9 0
PLR High(>=158.15) Low(<158.15)
p = 0.38
High(>=158.15) 49 30 12 4 2
Low(<158.15) 51 33 16 7 0

Fig. S2a

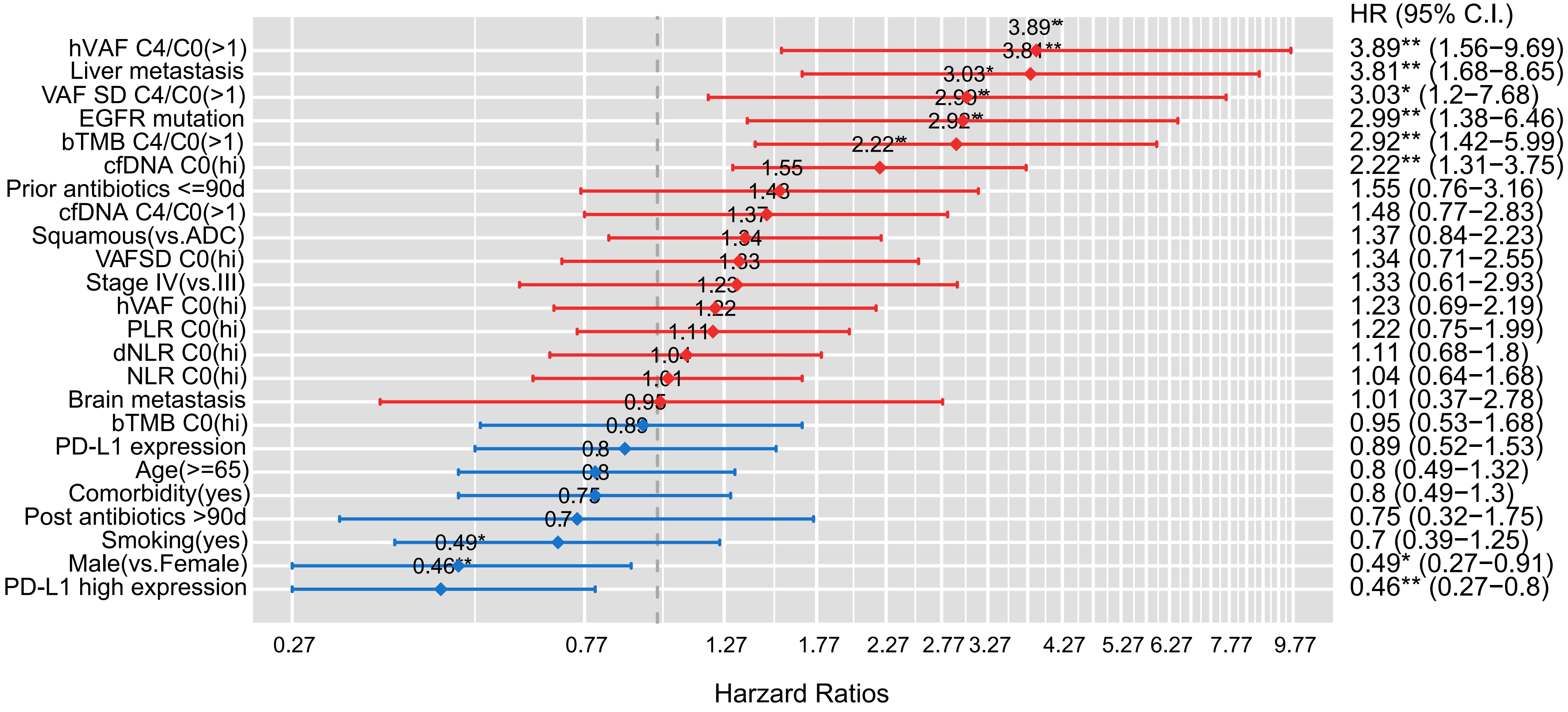
HR (95% C.I.)
hVAF C4/C0(>1)
Liver metastasis
VAF SD C4/C0(>1)
EGFR mutation
bTMB C4/C0(>1)
cfDNA C0(hi)
Prior antibiotics <=90d
cfDNA C4/C0(>1)
Squamous(vs.ADC)
VAFSD C0(hi)
Stage IV(vs.III)
hVAF C0(hi)
PLR C0(hi)
dNLR C0(hi)
NLR C0(hi)
Brain metastasis
bTMB C0(hi)
PD-L1 expression
Age(>=65)
Comorbidity(yes)
Post antibiotics >90d
Smoking(yes)
Male(vs.Female)
PD-L1 high expression
3.89** (1.56−9.69)
3.81** (1.68−8.65)
3.03* (1.2−7.68)
2.99** (1.38−6.46)
2.92** (1.42−5.99)
2.22** (1.31−3.75)
1.55 (0.76−3.16)
1.48 (0.77−2.83)
1.37 (0.84−2.23)
1.34 (0.71−2.55)
1.33 (0.61−2.93)
1.23 (0.69−2.19)
1.22 (0.75−1.99)
1.11 (0.68−1.8)
1.04 (0.64−1.68)
1.01 (0.37−2.78)
0.95 (0.53−1.68)
0.89 (0.52−1.53)
0.8 (0.49−1.32)
0.8 (0.49−1.3)
0.75 (0.32−1.75)
0.7 (0.39−1.25)
0.49* (0.27−0.91)
0.46** (0.27−0.8)
0.27
0.77
1.27
1.77
2.27
2.77
3.27
4.27
5.27
6.27
7.77
9.77
Harzard Ratios

Fig. S2b

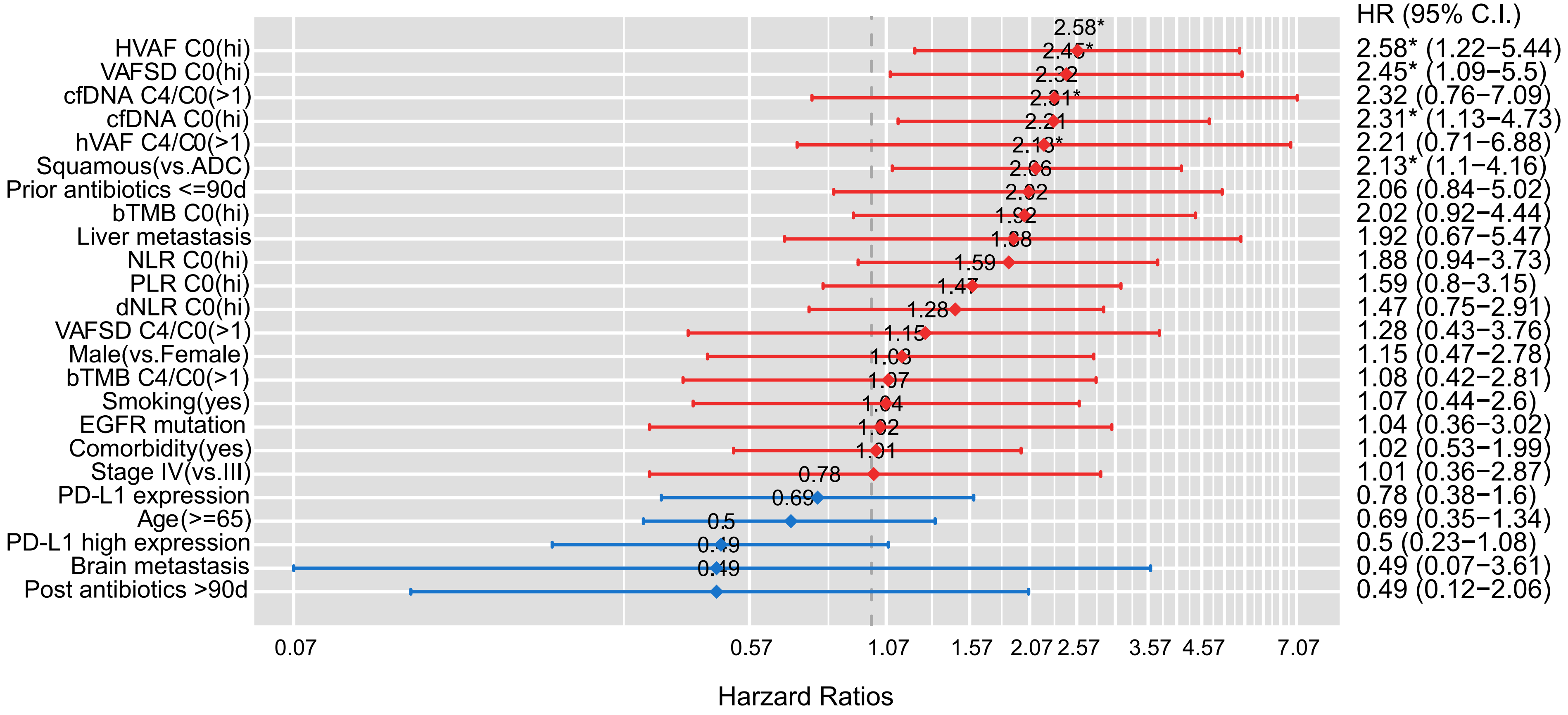
HR (95% C.I.)
HVAF C0(hi)
VAFSD C0(hi)
cfDNA C4/C0(>1)
cfDNA C0(hi)
hVAF C4/C0(>1)
Squamous(vs.ADC)
Prior antibiotics <=90d
bTMB C0(hi)
Liver metastasis
NLR C0(hi)
PLR C0(hi)
dNLR C0(hi)
VAFSD C4/C0(>1)
Male(vs.Female)
bTMB C4/C0(>1)
Smoking(yes)
EGFR mutation
Comorbidity(yes)
Stage IV(vs.III)
PD-L1 expression
Age(>=65)
PD-L1 high expression
Brain metastasis
Post antibiotics >90d
2.58* (1.22−5.44)
2.45* (1.09−5.5)
2.32 (0.76−7.09)
2.31* (1.13−4.73)
2.21 (0.71−6.88)
2.13* (1.1−4.16)
2.06 (0.84−5.02)
2.02 (0.92−4.44)
1.92 (0.67−5.47)
1.88 (0.94−3.73)
1.59 (0.8−3.15)
1.47 (0.75−2.91)
1.28 (0.43−3.76)
1.15 (0.47−2.78)
1.08 (0.42−2.81)
1.07 (0.44−2.6)
1.04 (0.36−3.02)
1.02 (0.53−1.99)
1.01 (0.36−2.87)
0.78 (0.38−1.6)
0.69 (0.35−1.34)
0.5 (0.23−1.08)
0.49 (0.07−3.61)
0.49 (0.12−2.06)
0.07
0.57
1.07
1.57
2.07
2.57
3.57
4.57
7.07
Harzard Ratios

Fig. S2c

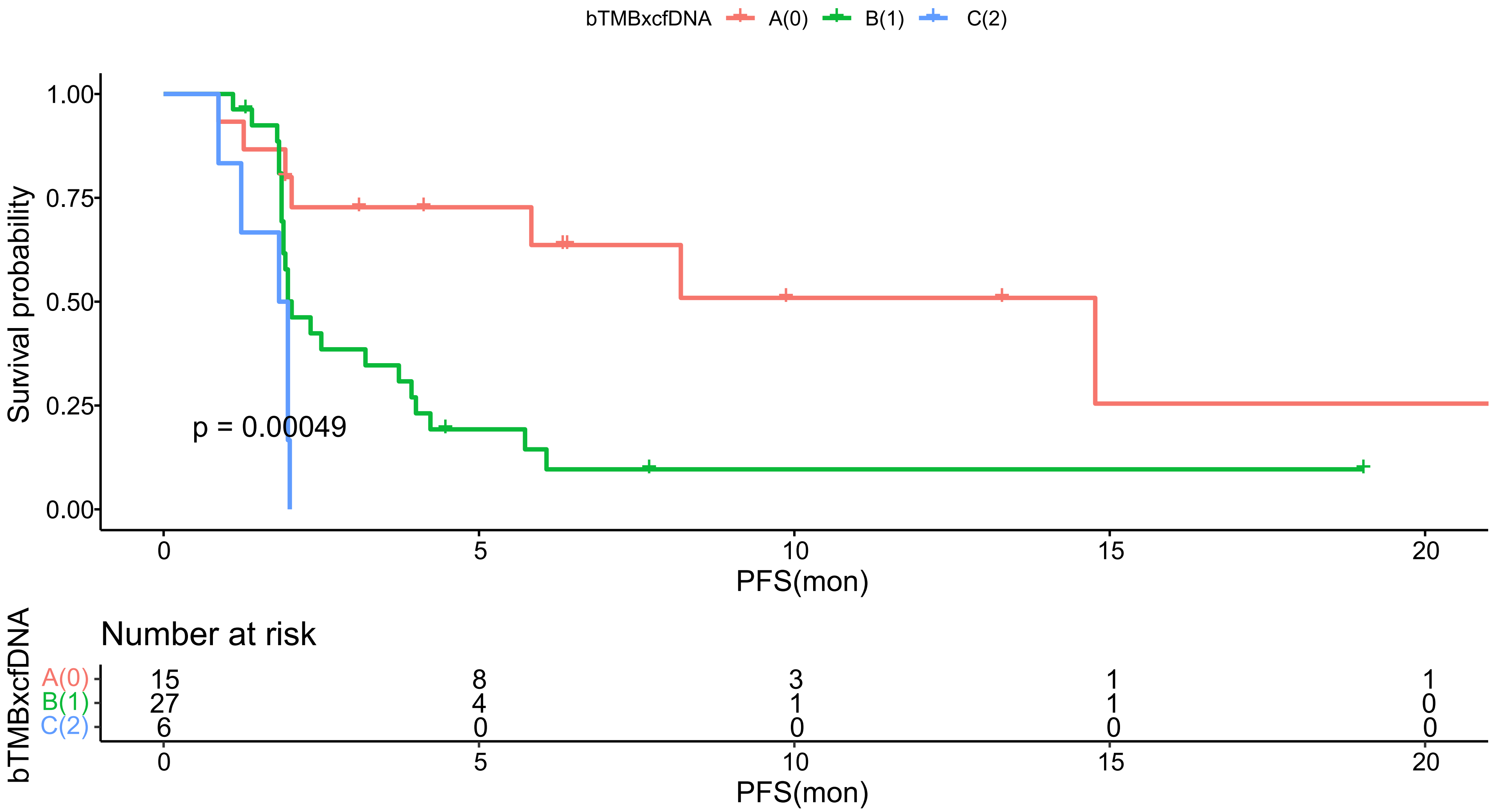

**Score group**	**bTMB C0 to C4**		**cfDNA C0 (ng/mL)**		**HR (95% CI)**	**P**
	Dec or NC(0)	Inc(1)	<8.6(0)	≥8.6(1)		
**A(0)**	0		0		1 (ref)	<0.001
**B(1)**	1		0		3.28 (1.37-7.87)	
	0		1			
**C(2)**	1		1		8.98 (2.73-29.56)	

Fig. S2d

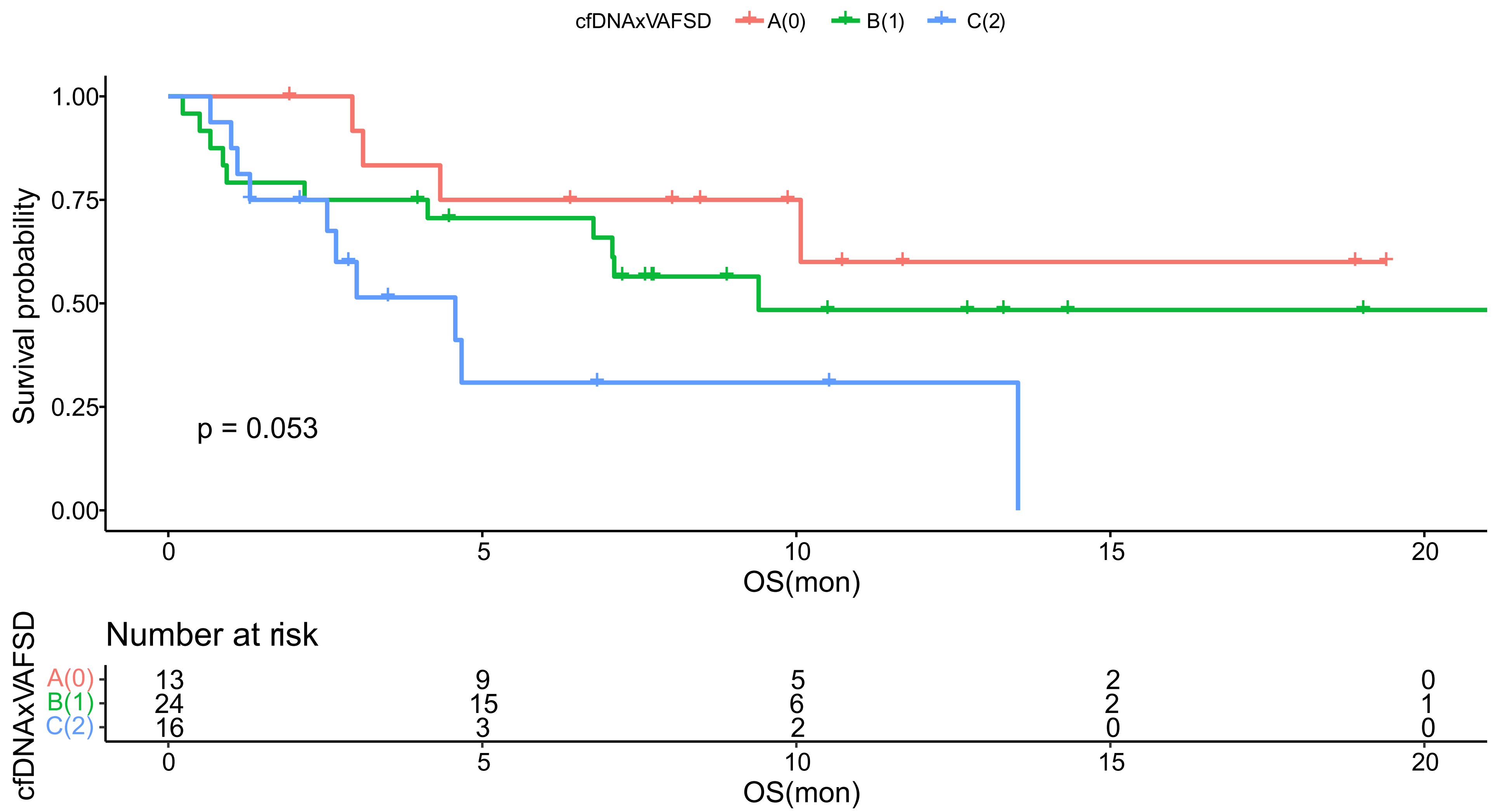

Score group	cfDNA C0 (ng/mL)		VAFSD C0		HR (95% CI)	P
	<8.6(0)	≥8.6(1)	<0.014(0)	≥0.014(1)		
**A(0)**	0		0		1 (ref)	0.053
**B(1)**	1		0		1.75 (0.56-5.50)	
	0		1			
**C(2)**	1		1		3.67 (1.13-11.88)	
